# A randomized, double-blind, placebo-controlled trial of IL-7 in critically ill patients with COVID-19

**DOI:** 10.1172/jci.insight.189150

**Published:** 2025-02-04

**Authors:** Manu Shankar-Hari, Bruno Francois, Kenneth E. Remy, Cristina Gutierrez, Stephen Pastores, Thomas Daix, Robin Jeannet, Jane Blood, Andrew H. Walton, Reinaldo Salomao, Georg Auzinger, David Striker, Robert S. Martin, Nitin J. Anand, James Bosanquet, Teresa Blood, Scott Brakenridge, Lyle L. Moldawer, Vidula Vachharajani, Cassian Yee, Felipe Dal-Pizzol, Michel Morre, Frederique Berbille, Marcel van den Brink, Richard Hotchkiss

**Affiliations:** 1 Department of Translational Critical Care Medicine, Centre for Inflammation Research, Institute for Regeneration and Repair, The University of Edinburgh, Scotland, United Kingdom.; 2 Medical-Surgical ICU & Inserm CIC 1435 Centre Hospitalier Universitaire, Limoges, France.; 3 Department of Medicine, Washington University in St. Louis, School of Medicine, St. Louis, Missouri, USA.; 4 Department of Critical Care Medicine, MD Anderson Cancer Center, Houston, Texas, USA.; 5 Department of Critical Care Medicine, Memorial Sloan Kettering Cancer Center, New York, New York, USA.; 6 Department of Anesthesiology, Washington University in St. Louis, School of Medicine, St. Louis, Missouri, USA.; 7 Department of Medicine, Universidade Federal de Sao Paulo (Unifesp), Sao Paulo, Brazil.; 8 Department of Intensive Care Medicine, King’s College Hospital, London, United Kingdom.; 9 Department of Critical Care Medicine, Missouri Baptist Medical Center, St. Louis, Missouri, USA.; 10 Department of Surgery, Sepsis and Critical Illness Research Center, University of Florida College of Medicine, Gainesville, Florida.; 11 Department of Critical Care Medicine, Cleveland Clinic, Cleveland, Ohio, USA.; 12 Department of Medicine, Hospital Sao Jose, Criciuma, Santa Catarina, Brazil.; 13 RevImmune, Paris, France.; 14 City of Hope, Los Angeles, California, USA.

**Keywords:** COVID-19, Clinical trials, Infectious disease, Immunotherapy

## Abstract

BACKGROUND. Lymphopenia and failure of lymphocytes to mount an early IFN-γ response correlate with increased mortality in COVID-19. Given the essential role of CD4 helper and CD8 cytotoxic cells in eliminating viral pathogens, this profound loss in lymphocytes may impair patients’ ability to eliminate the virus. IL-7 is a pleiotropic cytokine that is obligatory for lymphocyte survival and optimal function.

METHODS. We conducted a prospective, double-blind, randomized, placebo-controlled trial of CYT107, recombinant human IL-7, in 109 critically ill, patients with lymphopenia who have COVID-19. The primary endpoint was to assess CYT107’s effect on lymphocyte recovery with secondary clinical endpoints including safety, ICU and hospital length-of-stay, incidence of secondary infections, and mortality.

RESULTS. CYT107 was well tolerated without precipitating a cytokine storm or worsening pulmonary function. Absolute lymphocyte counts increased in both groups without a significant difference between CYT107 and placebo. Patients with COVID-19 receiving CYT107 but not concomitant antiviral medications, known inducers of lymphopenia, had a final lymphocyte count that was 43% greater than placebo (P = 0.067). There were significantly fewer treatment-emergent adverse events in CYT107 versus placebo-treated patients (P < 0.001), consistent with a beneficial drug effect. Importantly, CYT107-treated patients had 44% fewer hospital-acquired infections versus placebo-treated patients (P = 0.014).

CONCLUSION. Given that hospital-acquired infections are responsible for a large percentage of COVID-19 deaths, this effect of CYT107 to decrease nosocomial infections could substantially reduce late morbidity and mortality in this highly lethal disease. The strong safety profile of CYT107 and its excellent tolerability provide support for trials of CYT107 in other potential pandemic respiratory viral infections.

TRIAL REGISTRATION. NCT04379076, NCT04426201, NCT04442178, NCT04407689, NCT04927169.

FUNDING. Funding for the trial was provided by RevImmune and the Cancer Research Institute.

## Abstract 2

COVID-19 patients were treated with IL-7. IL-7 was well tolerated and decreased secondary infections. Lymphocyte counts increased in treated and control groups.

## Graphical abstract

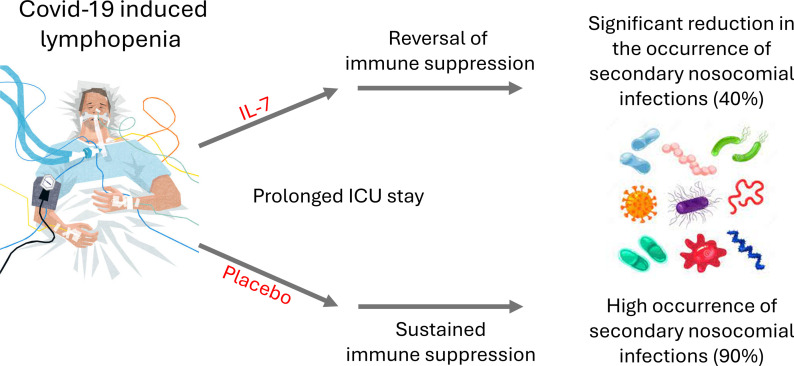

## Introduction

Currently, the predominant paradigm regarding the pathophysiology of COVID-19 is an excessive, hyperinflammatory, cytokine-mediated response leading to severe organ injury (1–3). A number of therapies have been demonstrated to improve morbidity and mortality in patients with COVID-19 — e.g., corticosteroids, Janus kinase inhibitors, and IL-6 antagonists (4). These therapies act to attenuate the host immune response, which is consistent with the understanding that a damaging cytokine response is the central driving force in the disorder.

Although an overexuberant inflammatory cytokine-mediated response likely plays a fundamental role in the COVID-19 disease process, a seemingly paradoxical alternative mechanism for the morbidity and mortality occurring in patients with COVID-19 is immunosuppression with resultant failure of the host to mount an effective antiviral response (5–9). Most of the deaths due to COVID-19 occur in elderly patients with immunosenescence and/or patients with serious comorbidities, 2 patient groups that often fail to mount a robust immune response to invading pathogens. Additional data supportive of the contention that failure of host immunity is a critical factor in morbidity and mortality of COVID-19 is provided by studies showing a strong relationship between SARS-CoV-2 viral load and increased disease severity and mortality (10, 11). Importantly, autopsy studies of patients who died of COVID-19 demonstrate viral persistence in lungs and other organs with viral inclusion bodies and extremely elevated levels of SARS-CoV-2 as detected by qPCR (12–15). These postmortem studies showing persistence of high levels of the virus systemically are consistent with a failure of the host to eliminate the invading virus and that unchecked viral replication is likely contributing to morbidity and mortality. Impaired host immunity may also be a factor in the high incidence of secondary hospital-acquired infections, which occur in many critically ill patients with COVID-19 and which are often the more proximate cause of death (16–19).

One of the earliest and most common reported defects in host immunity in patients with COVID-19 is lymphopenia (20). Autopsy studies of patients who died of COVID-19 showed that the decrease in circulating lymphocytes was accompanied by a corresponding depletion of tissue lymphocytes (12). In addition to a decrease in the number of lymphocytes, there is also a concomitant decline in lymphocyte function as indicated by impaired production of IFN-γ, a cytokine that plays a key role in host defenses (5, 9, 21). Importantly, both lymphopenia and the failure of T cells to mount an IFN-γ response correlate with an increase in fatal outcomes in patients with COVID-19 (20, 21). Given the essential role of CD4 helper T cells and CD8 cytotoxic T cells in eliminating viral pathogens, it is likely that the profound loss in the number and function of lymphocytes is impairing the ability of the host to eliminate the virus (22–24).

IL-7 is a pleiotropic, antiapoptotic cytokine that is required for lymphocyte survival and expansion (25, 26). IL-7 reverses T cell exhaustion, a common immunologic abnormality occurring in viral infections such as COVID-19 (5, 22). Animal studies have shown that IL-7 is required for the optimal response to acute influenza infection, since it shapes the early priming of CD8 cytotoxic T cells (27). Prophylactic administration of IL-7 protects against lethal influenza in a murine model (27). IL-7 immune therapy has also shown the ability to decrease viral load and ameliorate organ injury in other viral infectious models (28).

Although not yet clinically approved, CYT107 — a glycosylated, long-acting, recombinant human IL-7 — has been used in over 650 adult and pediatric patients with severe lymphopenia due to various etiologies including sepsis, AIDS, radiation and chemotherapy, bone marrow reconstitution following stem cell transplantation, and refractory viral and fungal infections (29–32). In patients with septic shock and lymphopenia, CYT107 caused a 3- to 4-fold increase in circulating CD4 and CD8 T cells as well as inducing T cell proliferation and activation (31). In clinical trials of patients with HIV infection who had lymphopenia despite optimal antiretroviral therapy, CYT107 caused a 2- to 4-fold dose-dependent increase in circulating CD4 and CD8 T cells (32). CYT107-treated patients consistently demonstrate increased circulating lymphocyte counts and tolerate the drug well, with injection-site reactions being the only serious adverse effect. Importantly, we previously described the compassionate use of CYT107 in 12 critically ill, mechanically ventilated patients with COVID-19 with profound lymphopenia (33). The study showed that CYT107 was well tolerated without exacerbating inflammation or aggravating lung injury and was associated with a more robust return of lymphocytes to normal levels compared with a matched control population.

The current study reports the results of a prospective, randomized, double-blind, multicenter, placebo-controlled trial of CYT107 in 109 critically ill patients with COVID-19 with lymphopenia. The primary endpoint of the study was to determine if CYT107 was more effective in restoring absolute lymphocyte counts (ALCs) versus placebo. An additional key study objective was to determine if CYT107 could be safely used in patients with COVID-19 with severe respiratory failure without leading to further respiratory compromise or exacerbating systemic inflammation. Other aims of the study were to evaluate the effects of CYT107 on major clinical outcomes, including ICU and hospital length of stay, incidence of secondary hospital-acquired infections, and mortality as potentially indicative of putative beneficial effects of immune adjuvant therapy in this patient population with severe viral pneumonia and lymphopenia.

## Results

**Figure 1:**
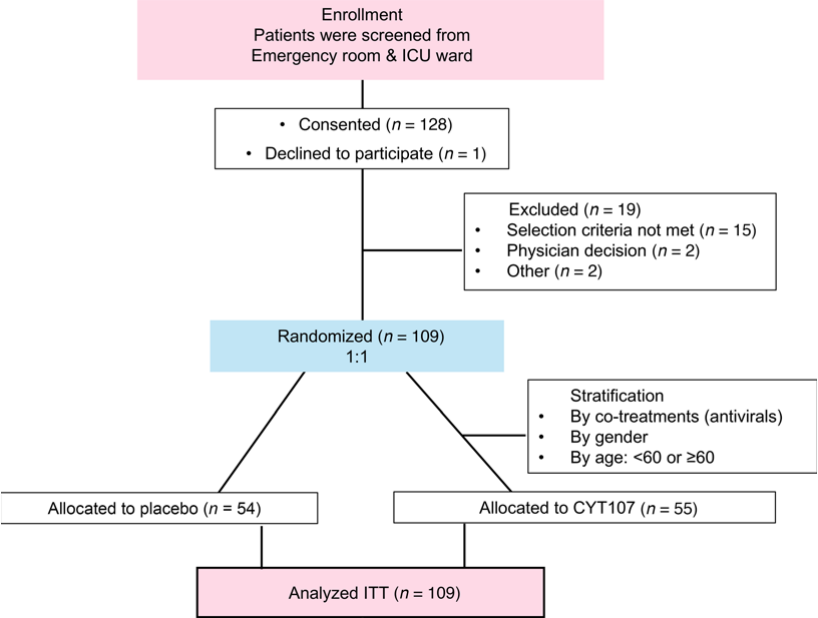
Trial enrollment details. Details on patient enrollment, allocation, stratification, and number analyzed are presented. Hospitalized patients with respiratory distress who were positive for SARS-CoV-2 were identified. Patients were further screened for eligibility as per inclusion and exclusion criteria.

A total of 109 patients were enrolled in 4 different countries (Figure 1 and Supplemental Figure 1; supplemental material available online with this article; https://doi.org/10.1172/jci.insight.189150DS1) between May of 2020 and February of 2022. There were no significant differences between the CYT107 group and the placebo group in patient age, sex, BMI, race, comorbidities, WHO or NEWS-2 clinical scores, C-reactive protein, ferritin, or D-Dimer at enrollment (Table 1). There was also no difference in baseline patient values for complete WBC count, absolute neutrophil count, or absolute monocyte at trial initiation; there was a small but statistically significant difference in baseline ALCs (Supplemental Table 1). The average number of doses of CYT107 received by patients was 3.29 doses (Supplemental Figure 2). In total, 62% of the patients enrolled were being mechanically ventilated at baseline or during the study. CYT107 or placebo were discontinued in 75 patients due to hospital discharge or death. Study enrollment was halted at 109 patients out of a planned enrollment of 220 patients because of difficulty in recruiting additional patients with the decline of the COVID-19 pandemic.

### Lymphocyte counts increased in both CYT107- and placebo-treated patients and were not different.

The effect of CYT107 on the ALC was determined using a mixed model of repeated measures starting at multiple time points after initiation of CYT107 until day 30 or at hospital discharge, if occurring before day 30 (34). The last ALCs in the CYT107- and placebo-treated patients were 1,335 (±136) and 1,125 (±87) cells/μL (mean ± SEM), respectively, and were not statistically different (Table 2). Importantly, 60% of patients with COVID-19 receiving CYT107 were also receiving antiviral nucleoside analogs, predominantly remdesivir (Supplemental Table 2). Lymphopenia is a well-known side effect of this class of drugs (35–37). Given this potential effect of antiviral therapy to inhibit CYT107’s ability to induce lymphocyte proliferation and to increase lymphocyte counts, the effect of CYT107 on ALCs was also analyzed separately in CYT107- and placebo-treated patients who did not receive concurrent antiviral therapy. Patients with COVID-19 who received CYT107 without concomitant nucleoside analogs had a final ALC that was 43% greater than placebo-treated patients — i.e., 1,624 (±228) versus 1,136 (±119) cells/μL (SEM) respectively (*P* = 0.067).

### CYT107 was safe and did not exacerbate organ dysfunction.

CYT107 was well tolerated, and surprisingly, there were fewer overall treatment emergent adverse events (TEAEs) recorded in the CYT107 treated patients versus placebo — i.e., 195 versus 396 TEAEs, respectively (*P* < 0.0001) (Table 3). The CYT107 group also experienced statistically fewer adverse events typical of COVID pathology such as renal (64% decrease), cardiac (45% decrease), central nervous system (81% decrease), and respiratory (43% decrease) (all *P* < 0.05) (Table 3). One patient in the study developed a fever and had increased respiratory distress requiring intubation and mechanical ventilation after the second dose of the study drug. The treatment was halted, and unblinding showed that the patient was receiving CYT107. The patient rapidly improved over the next 12–24 hours and was taken off the ventilator. The patient recovered uneventfully and was discharged from the ICU and subsequently from the hospital. Following this event, the Data Safety Monitoring Board (DSMB) met and reviewed all patients in the study to date. After the review, the DSMB recommended restarting the study without any protocol modifications. The study was continued with no further similar episodes noted.

### CYT107 did not induce cytokine storm, as it had no effect on circulating IL-6, TNF-α, or IL-10 cytokine levels.

**Figure 2:**
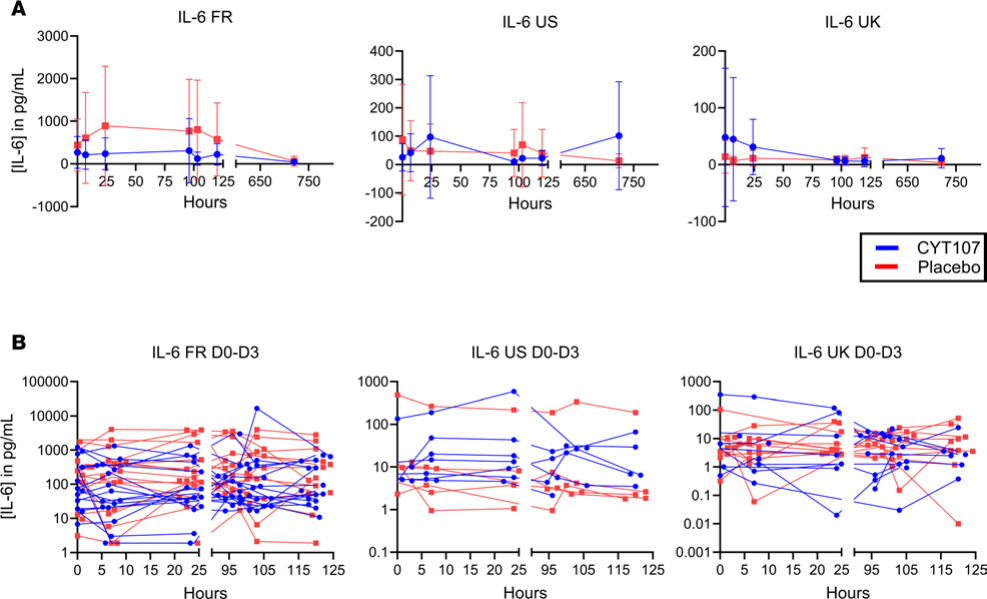
CYT107 did not increase circulating levels of IL-6. Blood samples were collected from patients at 6–7 hours and 20–22 hours after the first and second doses of CYT107 or placebo. (**A** and **B**) Results showed that circulating levels of IL-6 were not different in patients receiving CYT107 versus placebo and represent averaged data (**A**) and individually plotted data (**B**). See Supplemental Figures 3 and 4 for results for IL-10 and TNF-α. Data are shown as mean ± SEM. FR, France; US, United States, UK, United Kingdom; D0, day 0, D3, day 3.

To determine the potential effect of CYT107 to affect levels of proinflammatory or antiinflammatory cytokines, blood samples were collected at 6–7 hours and 20–22 hours after administration of the first 2 doses of CYT107 or placebo from approximately 60% of the patients. Results showed that CYT107 had no significant effect on circulating levels of IL-6, TNF-α, or IL-10 versus placebo (Figure 2 and Supplemental Figures 3 and 4).

### Patients receiving CYT107 trended to shorter ICU length of stays versus placebo-treated patients.

**Figure 3:**
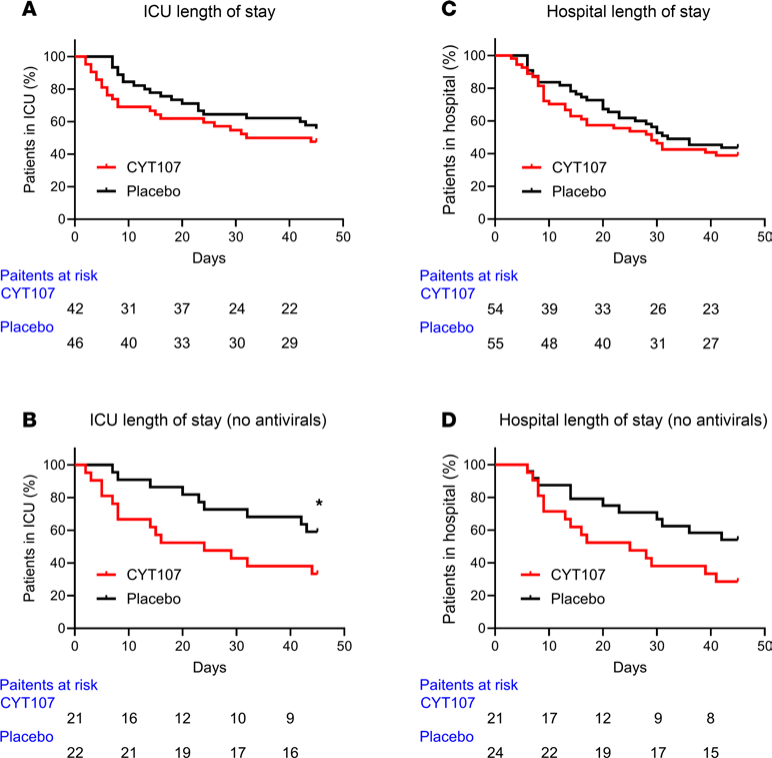
Effects of CYT107 on ICU and hospital length of stay. (**A**) Comparison of the number of ICU days in the CYT107 versus placebo group showed that there was no difference in the number of ICU days in the patients receiving CYT107 versus placebo — i.e., 31 days versus 45 days respectively (*P* = 0.3366) . Because of potential effects of antiviral agents to inhibit the putative beneficial effects of CYT107, the number of ICU days were also calculated for patients receiving CYT107 or but no antiviral drugs versus placebo treated patients who were not receiving antiviral drugs. (**B**) Patients receiving CYT107 but no antiviral medications did have a statistically significant decrease in their number of ICU days — i.e., 24 days versus 45 days respectively (*P* = 0.0482). (**C**) Results for hospital length of stay showed similar trends as ICU length of stay. Hospitalization days in patients receiving CYT107 versus placebo — i.e., 29 days versus 45 days, respectively (*P* = 0.4096). (**D**) Comparison of the days of hospitalization in CYT107-treated patients who were not receiving antiviral agents was close to statistical significance at 28 days versus 45 days for CYT107- and placebo-treated patients respectively (*P* = 0.0623). Survival, ICU, and hospital length of stay were analyzed with a log-rank (Mantel-Cox) test to compare the curves. Note that patients who died during their ICU or hospital stay were censored at day 45 even if they died prior to that day. Comparison of ICU and hospital length of stay was performed using the log-rank (Mantel-Cox) test. *P < 0.05.

Comparison of the number of ICU days in the CYT107 versus the placebo group showed that there was no difference in the number of ICU days in the patients receiving CYT107 versus placebo — i.e., 31 days versus 45 days respectively (*P* = 0.3366) (Figure 3A). Because of potential effects of antiviral agents to inhibit the putative beneficial effects of CYT107, the number of ICU days was also calculated for patients receiving CYT107 or placebo but receiving no additional antiviral drugs. Patients receiving CYT107 but no antiviral medications had a statistically significant decrease in their number of ICU days versus placebo-treated patients not receiving antivirals — i.e., 24 days versus 45 days respectively (*P* = 0.0482) (Figure 3B).

### Patients receiving CYT107 trended toward shorter hospital length of stays versus placebo-treated patients.

Results for hospital length of stay showed similar trends as ICU length of stay. Hospitalization days in patients receiving CYT107 versus placebo were 29 days versus 45 days, respectively (*P* = 0.4096) (Figure 3C). Comparison of the days of hospitalization in CYT107 versus placebo-treated patients who were not receiving antiviral agents was close to statistical significance at 28 days versus 45 days for CYT107- and placebo-treated patients, respectively (*P* = 0.0623) (Figure 3D).

### Patients receiving CYT107 had fewer secondary infections versus placebo-treated patients.

The development of hospital-acquired secondary infections was evaluated by 2 groups of a 3-member panel of experienced intensive care physicians using the Center for Disease Control criteria for healthcare-associated infections (https://www.cdc.gov/healthcare-associated-infections/index.html) as previously described (31) (Supplemental Methods). The panel members were blinded to the patient identity and treatment group. Data show that 28 of 55 (50.9%) versus 49 of 54 (90.7%) of CYT107-treated and placebo-treated patients, respectively, developed secondary infections, (*P* = 0.013) (Table 4). The particular pathogens that were identified as the cause of the secondary hospital-acquired infection in each individual patient include many opportunistic type organisms (Table 5).

### There was no difference in all-cause mortality in CYT107- versus placebo-treated patients.

**Figure 4:**
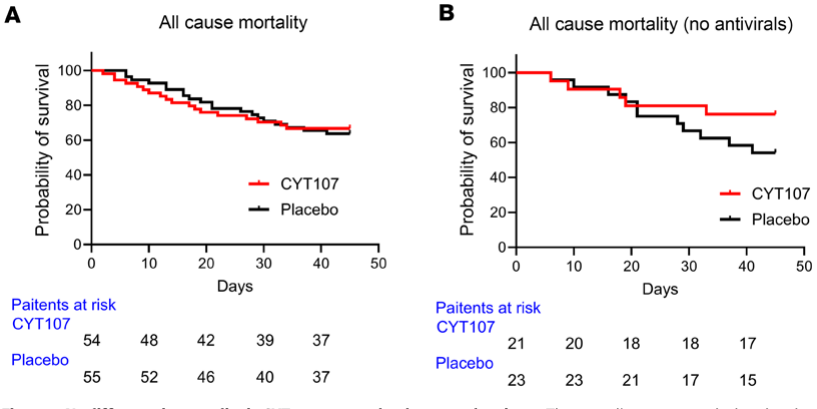
No difference in mortality in CYT107- versus placebo-treated patients. The mortality rate was calculated at day 30 or hospital discharge if patients left the hospital prior to day 30. (**A**) The overall mortality was 32.7% in CYT107-treated patients (*n* = 55) versus 38.9% in placebo-treated patients (*n* = 54) and was not statistically different (*P* = 0.59) (Table 3). (**B**) Because of potential effects of antiviral agents to inhibit the putative beneficial effects of CYT107, mortality rates were also determined separately for patients receiving CYT107 but no antiviral drugs and similarly for placebo-treated patients who were not receiving antiviral drugs. Mortality for CYT107-treated patients (*n* = 22) and placebo-treated patients (*n* = 23) was 22.7% and 47.8%, respectively (*P* = 0.17) (Table 3). Comparison of mortality was performed using the log-rank (Mantel-Cox) test.

The mortality rate was calculated at day 30 or hospital discharge, if patients left the hospital prior to day 30. The all-cause mortality was 32.7% in CYT107-treated patients (*n* = 55) versus 38.9% in placebo-treated patients (*n* = 54) and was not statistically different (*P* = 0.59) (Figure 4A and Table 2). The individual patient cause of death as assigned by the treating physicians and then validated by pharmacovigilance is provided in Supplemental Table 3. Because of potential effects of antiviral agents to inhibit the putative beneficial effects of CYT107 on lymphocyte proliferation, all-cause mortality was also determined separately for patients who received CYT107 or placebo alone (no antiviral drugs). All-cause mortality in these patients was 22.7% (*n* = 5/22) and 47.8% (*n* = 11/23) for CYT107 and placebo groups respectively (*P* = 0.17) (Figure 4B and Table 3). The mortality rate for patients being treated with both CYT107 and antiviral nucleoside inhibitors was 39.4% (*n* = 13/33), while the mortality rate for patients receiving CYT107 alone (no antivirals drug therapy) was 22.7% (*n* = 5/22) (Figure 4B and Table 2). The mortality rate for patients being treated with both antiviral nucleoside inhibitors and CYT107 or placebo were respectively 39.4% (*n* = 13/33) and 32.3% (10/31) (*P* = 0.94; not significant) (Table 3).

## Discussion

Our study has particular importance, since it represents one of the few clinical trials to test drug therapies that enhance rather than inhibit the host immune response in patients with COVID-19. Results demonstrated that recombinant human IL-7 (CYT107), which potently enhances adaptive immunity, did not precipitate a cytokine storm and was safe and well tolerated.

### CYT107 clinical tolerability and safety.

An important finding of the present study was the excellent clinical tolerability of CYT107 in critically ill patients with COVID-19. Patients receiving IL-7 did have a highly statistically significant decrease in TEAEs — i.e., 195 versus 396 TEAEs, respectively (*P* < 0.0001). Many of these adverse events directly reflect COVID-19 pathology (Table 3). Therefore, this significant decrease in TEAEs is consistent with a putative beneficial effect of IL-7 in patients with COVID-19. With the exception of a single patient who had a transient reversible respiratory deterioration, there was no evidence that CYT107 worsened pulmonary function, precipitated hemodynamic instability, or increased levels of proinflammatory cytokines (Table 3, Figure 2, and Supplemental Figures 3 and 4). Specifically, circulating levels of IL-6, TNF-α, and IL-10 were not different in CYT107- versus placebo-treated patients after drug administration (Figure 2 and Supplemental Figures 3 and 4). Interestingly, the levels of IL-6 and IL-10 were higher in the laboratory testing center in France compared with the United Kingdom and United States testing sites (Figure 2 and Supplemental Figures 3 and 4). Potential reasons for this site variation include different reference standards used for instrument calibration, differences in aliquoting and freezing after blood draw, time of storage, and storage conditions. Importantly, there were no differences in the effect of CYT107 versus placebo to increase cytokine levels at the 3 independent testing sites (38, 39).

The present results showing the safety of CYT107 in this patient population with severe COVID-19 are in agreement with a previous, smaller study of 12 mechanically ventilated critically ill patients with COVID-19 treated with CYT107 (33). In the previous study, CYT107 was well tolerated without inducing changes in temperature, blood pressure, or PaO_2_/FiO_2_ ratios. As in the present study, there were no detectable adverse clinical effects of CYT107 on any organ system. Increased clotting disorders including pulmonary emboli and deep venous thrombosis are another common complication of COVID-19. In that regard, there was no increase in the incidence of venous thrombosis in the CYT107-treated group versus placebo. Furthermore, the day 30 D-Dimer levels in CYT107- versus placebo-treated patients were similar in the present trial — i.e., 3,041.7 ± 7478.9 versus 3,088.2 ± 3749.9 ng/mL, respectively. It is important to note that, because of requirements to verify that patients met the inclusion and exclusion criteria (e.g., decreased ALCs) and the necessity of obtaining informed consent, CYT107 was typically not administered until 48–72 hours after hospital admission. This delay in administering CYT107 is important because studies in patients with sepsis show that levels of proinflammatory cytokines fall precipitously during the first 48–72 hours and are much reduced compared with their peak concentrations. Thus, this delay in administration of CYT107 makes it unlikely that CYT107 was administered during the early more inflammatory phase of COVID-19. It is also important to note that CYT107 was administered i.m. resulting in a slower uptake and better tolerability compared with i.v. administration (40). One likely explanation for the safety of CTY107 relates to the unique properties of the IL-7 receptor. Once activated by IL-7, the IL-7 receptor is internalized and nonresponsive to additional doses of IL-7 for 48–72 hours (41). In addition, as T cells become more activated, IL-7 receptor expression decreases, making IL-7 unable to overstimulate the activated lymphocytes.

### Protection from hospital-acquired secondary infections.

Although patients with COVID-19 may die during the initial hyperinflammatory cytokine storm–mediated phase, many patients survive this early phase but subsequently develop nosocomial bacterial and fungal infections, which are a major cause of morbidity and mortality (14–17). Unresolving secondary pneumonia has been linked to death in patients with COVID-19 (42). The high incidence of hospital-acquired infections in patients with COVID-19 is likely due in part to the effect of SARS-CoV-2 to weaken essential components of the immune system, including by inducing profound lymphopenia, which has been associated with increased risk of morbidity and mortality in patients with sepsis and COVID-19 (6, 18, 19, 24, 43–45). IL-7 increases both the number as well as the functional activity of CD4 helper T and CD8 cytotoxic T lymphocytes that play a critical role in antiviral host defenses (27, 28, 32, 46, 47). The present results showing a major decrease in secondary infections in CYT107-treated patients with COVID-19 are consistent with results of an earlier encouraging trial of CYT107 in 12 critically ill patients with COVID-19 in which secondary infections occurred in 7 (58%) patients with COVID-19 treated with CYT107 versus 11 of 13 (85%) historical control COVID-19 control patients (33).

### Potential mechanisms for the effect of CYT107 to decrease secondary infections.

IL-7 has been termed the “maestro of the immune system” because of its myriad effects to orchestrate host immunity (25). Consequently, there are several potential mechanisms whereby CYT107 could have led to a decrease in secondary infections in critically ill patients with COVID-19. Despite the absence of a significant increase in circulating lymphocyte numbers, there is compelling historical evidence to suggest that CYT107 administration affects lymphocyte function and host protective immunity that would result in the more rapid containment and elimination of the invading SARS-CoV-2 (5, 22, 47–49). Early control of the initial viral infection could prevent secondary bacterial or fungal infections (17, 18, 20, 21). IL-7 plays a critical role in shaping T cell responses to respiratory viral infections by increasing both the number and function of viral antigen–specific T cells including CD8 cytotoxic T cells (29–31, 50, 51). The importance of an early robust antiviral antigen-specific T cell response in COVID-19 is highlighted by Tan and colleagues who reported that the early induction of functional SARS-CoV-2–specific T cells was associated with mild disease and more rapid viral clearance (21). Similarly, Sattler et al. observed that patients with COVID-19 who had a blunted T cell response to SARS-CoV-2 cell membrane, nucleocapsid, or spike protein antigens were more likely to die compared with patients with COVID-19 who did mount a robust T cell response (48). Importantly, we previously reported that therapy with CYT107 in a critically ill patient with COVID-19 with lymphopenia and poor T cell response to SARS-CoV-2 antigens resulted in an approximate 3-fold increase in the number of IFN-γ–producing lymphocytes that were reactive to the SARS-CoV-2 nucleocapsid and/or spike protein antigens (52). The increase in the number of CYT107-induced SARS-CoV-2 reactive T cells was temporally associated with rapid clinical improvement and recovery in the patient (49). IL-7 not only increases the number of T cells directed against SARS-CoV-2 viral antigens but also increases T cell receptor diversity, thereby potentially enhancing the intensity of the T cell response and lowering the risk of secondary infections (30, 32, 50). The trend toward shorter ICU days in patients with COVID-19 treated with CYT107 is consistent with an effect of CYT107 to accelerate viral clearance (Figure 3).

Additional mechanisms whereby IL-7 may act to decrease secondary nosocomial infections in patients with COVID-19 is its effect to reverse T cell exhaustion, which commonly occurs in patients with persistent viral infections including COVID-19 (51, 53). Investigators have reported that CD4 and CD8 T cells from patients with COVID-19 had expression of multiple inhibitory molecules including PD-1, PD-L1, Tim-3, and CTLA-4, indicative of T cell exhaustion (8, 21, 46, 51, 53). Importantly, investigators have demonstrated that ex vivo treatment of blood samples with IL-7 from patients with COVID-19 caused restoration in T cell proliferation and IFN-γ production (5, 22, 52, 54). Other mechanisms by which IL-7 could decrease secondary infections are its effects to activate innate lymphoid cells, including mucosally associated invariant T (MAIT) cells, which play a critical role in host defense against invading respiratory and gastrointestinal pathogens (47, 55). Studies by Hubrack and colleagues showed that MAIT cells from patients with COVID-19 have severely impaired function, which was reversed by ex vivo treatment with IL-7 (47). These investigators concluded that IL-7 treatment might be an effective method to protect against SARS-CoV-2 infections.

### CYT107’s typical effect to increase lymphocytes may have been inhibited by nucleoside analogs.

A surprising finding from the current study is the fact that both CYT107- and placebo-treated patients showed a similar increase in their ALCs. IL-7 is a lymphocyte growth factor that has unfailingly been demonstrated to cause a dose-dependent increase in CD4 and CD8 T cells in numerous clinical trials in oncology/hematology patients and in patients with severe infections (29, 31, 32, 50, 55, 56). Our previous trial of CYT107 in patients with COVID-19 showed that the group receiving CY107 had an ALC at day 30 that was more than 2-fold greater than the control group (33) Additionally, we previously reported that the ALC in a patient with COVID-19 who received CYT107 on a compassionate basis increased approximately 5–10 fold to a high of 5,100/μL, which is considerably greater than 3,500/μL, the upper limit of normal for ALCs (52). One potential reason for the lack of effect of CYT107 to increase patient lymphocyte counts compared with the placebo-treated group could be due to concomitant therapy with drugs that induce lymphopenia. Unlike previous CYT107 clinical studies in sepsis or COVID-19, 60% of patients treated with CYT107 in the present study received concurrent antiviral therapy with remdesivir or other drugs in its class that act as nucleoside analogs to inhibit nucleic acid polymerase (Supplemental Table 3). Inhibition of RNA or DNA polymerase leads to accumulation of toxic purine degradation by-products that can induce cell death (35–37). Lymphocytes are the cells that are most sensitive to these toxic purine degradation products, and lymphopenia is a well recognized complication of the use of this class of drugs (35–37).

### Implications of the present study regarding the underlying pathogenesis of COVID-19.

The findings from the present study have implications regarding the mechanisms responsible for morbidity and mortality in patients with COVID-19. Many investigators believe that most deaths in patients with COVID-19 are driven by an unbridled host immunoinflammatory response to the invading virus (1–3). These investigators argue that there is strong evidence of end-organ damage without viral invasion. Unfortunately, there has been a relative paucity of autopsy studies that investigated the link between organ injury and the presence of viral load, likely because of initial concerns regarding the transmissibility of SARS-CoV-2. However, several groups have reported the detection of high viral loads in lung in COVID-19 postmortem specimens (11–14). Menter et al. reported that postmortem examination of patients with COVID-19 showed high levels of SARS-CoV-2 as detected by quantitative PCR (qPCR) in the lungs of 20 of the 21 patients (12). Secondary superimposed bronchopneumonia was also present in 10 of the 21 patients underscoring the role of nosocomial infections. Similarly, Schaller et al. conducted autopsies on 10 patients with COVID-19 with severe acute respiratory distress syndrome and reported that SARS-CoV-2 was detectable by PCR in the respiratory tracts of all 10 patients (13). The most comprehensive postmortem study examining presence of SARS-CoV-2 in organ tissue samples was conducted by Stein and colleagues, who did complete autopsies on 44 patients who died of COVID-19 (14). Results showed that SARS-CoV-2 was widely distributed, predominantly in patients who died with severe COVID-19, and that virus replication was present in multiple respiratory and nonrespiratory tissues including the brain (14). Collectively, these studies are consistent with the concept that inability of the patients to successfully eradicate the invading SARS-CoV-2 results in ongoing viral-induced organ injury.

There is also increasing evidence that long COVID is associated with persistence of SARS-CoV-2 (57–59). Zuo and associates showed that tissue samples from a large cohort of patients who had seemingly recovered from COVID-19 had SARS-CoV-2 viral RNA present in multiple organs and blood for up to 4 months after infection (57). In addition, patients who had higher virus copy numbers were more likely to have symptoms consistent with long COVID. Findings from the present study do not directly address the critical question of whether the primary abnormality in COVID-19 is due to a damaging host immunoinflammatory response or a result of the failure of host immunity to contain SARS-CoV-2. However, the results showing that therapy with CYT107, a potent immune adjuvant, led to decreased secondary nosocomial infections supports the theory that failure to eliminate the SARS-CoV-2 is a key pathogenic mechanism underlying the ongoing morbidity and mortality.

### Limitations.

There are a number of limitations to the present investigation. One potential complicating factor is the evolution of SARS-CoV-2 during the study with the emergence of different strains of the virus with varying degrees of virulence. Secondly, the study included patients with COVID-19 from 4 different countries. Critical care management of patients such as respiratory ventilator support techniques were likely not identical at all sites. It is also important to note that the patients being treated with CYT107 in the present trial received fewer doses of the drug (average 3.29 doses) versus the typical 5–6 doses of CYT107 for the previous trials in sepsis and COVID-19. The lack of a more robust increase in the ALC is likely partly related to this shortened course of treatment. Different clinical management protocols could affect ICU and hospital outcomes. Finally, the relatively small number of patients included in the present trial prevents any firm conclusions regarding the potential clinical efficacy of CYT107 in patients with COVID-19.

### Conclusions.

In conclusion, the findings from this study have particular significance because they represent one of the few studies to test drugs that enhance patient immunity in critically ill patients with COVID-19. ALCs increased in both groups without a statistically significant difference between CYT107- and placebo-treated patients. This lack of an effect of CYT107 to increase the ALCs compared with placebo-treated patients, the primary endpoint of the study, may have been due in part to effects of the concomitant use of antiviral drugs, which can cause lymphopenia. Overall, CYT107 was well tolerated without any clinical or laboratory evidence of precipitating a cytokine storm or aggravating pulmonary inflammation in these patients with COVID-19. Specifically, there were significantly fewer treatment emergent adverse effects for most organ systems recognized as common targets of COVID-19 pathology, including the lung in the CYT107-treated group versus the placebo group. Moreover, CYT107 decreased secondary hospital-acquired infections by over 40% versus the placebo-treated group. Given that hospital-acquired infections are responsible for a large percentage of deaths in patients with COVID-19, this putative beneficial effect of CYT107 to decrease nosocomial infections could substantially reduce late morbidity and mortality in this highly lethal disease. The strong safety profile of CYT107 and its capacity to reduce secondary infections provide support for trials of CYT107 in other potential pandemic respiratory viral infections.

## Methods

### Sex as a biological variable.

The current study examined both male and female patients; similar results were found for both sexes.

### Study design and participants.

This prospective double-blind, randomized, placebo-controlled phase IIb trial of CYT107 was designed as a single study, but due to local regulatory constraints, it was conducted as individual cohorts in the United Kingdom, France, Brazil, and the United States using nearly identical protocols with minor exceptions (Supplemental Methods). At study end, the data from all cohorts were combined for a statistical analysis with a single statistical analysis plan (SAP).

The primary endpoint was an increase in the ALC with CYT107 evaluated in the context of an acceptable safety profile. Secondary endpoints were clinical outcomes including: clinical improvement defined as an improvement of the WHO 11-point ordinal scale from randomization through hospital discharge or day 30, number of organ support–free days during index hospitalization, incidence of secondary infections, number of days in the ICU and in the hospital during index hospitalization, and all-cause mortality through day 45 (60).

### Randomization and blinding.

Randomization assignment was assigned by the Electronic Data Capture system following data entry by the research team and informed consent with the investigator or PI approval for initiation of study drug treatment. A permuted-block randomization was used (1-CYT107 + 1 placebo) for an allocation ration of 1:1. Study drug or placebo were prepared at each site by the hospital pharmacy and delivered to the research team in a blinded fashion; members of the research team subsequently delivered them to ICU staff for administration to patients.

All study participants, clinical, and research teams were blinded to assignment of all but 1 study participant until after the final patient had completed treatment and all queries were resolved. There was 1 patient, as discussed above, where an emergency unblinding was required; this patient had been randomized to CYT107 and is included in all analyses.

### Study deviations.

IL-7 or placebo were discontinued in 75 patients due to hospital discharge or death. Study enrollment was halted at 109 patients out of a planned enrollment of 220 patients because of difficulty in recruiting additional patients as a result of the waning of the pandemic and rising costs of the trial. One patient who was randomized to placebo was treated with study drug.

### Inclusion criteria.

The main inclusion criteria were the following (a) hospitalized men and women aged ≥ 25–80 years of age; (b) COVID-19 diagnosed by any acceptable test available/utilized at each site; (c) 1 ALC ≤ 1,000 cells/mm^3^ collected at hospitalization or no more than 72 hours after admission; (d) hypoxemia requiring oxygen therapy at ≥ 2 L per minute nasal cannula or greater to keep saturations > 90%, noninvasive positive pressure ventilation (e.g., BIPAP), or intubation with mechanical ventilatory support for respiratory failure; and (e) signed informed consent by the patient or the patient’s legally authorized representative (Supplemental Methods).

### Exclusion criteria.

The main exclusion criteria were the following: (a) elevated liver function tests greater than 5 times upper limit of normal; (b) known or active autoimmune disease; (c) SOFA score ≥ 9 at baseline; (d) history of organ transplantation; and (e) patients with baseline Rockwood Clinical Frailty Scale ≥ 6 (Supplemental Table 1) (61, 62). Patients who were receiving antiviral therapies such as remdesivir, the IL-6 antagonist tocilizumab, and/or Janus kinase inhibitors were not excluded from enrollment.

### Demographic reporting.

Demographic identities were classified on hospital admission. This was typically performed by either the patient or a patient representative (next of kin, family member, etc.) during intake to the hospital on a form, which contained differing classifications and the option to write in a custom option. In the case of the patient being unresponsive and no viable representative being present, hospital staff would make initial determinations to be validated by patient/patient representatives at a later opportunity. No racial/ethnic classification was made for patients in the French cohort, as French law typically forbids collection of racial/ethnic identity in most cases.

### CYT107 dosing regimens.

Consistent with IL-7 receptor kinetic of reappearance (39), CYT107 was administered i.m. twice a week. Due to concerns that CYT107 might exacerbate the early hyperinflammatory phase of COVID-19, the first 12 patients who were randomized to receive CYT107 had an initial dose of 3 μg/kg of CYT107, followed by10 μg/kg of CYT107 for all subsequent doses. After DSMB confirmation that there was no evidence of any adverse effects in the these first 12 patients receiving the initial lower dose of CYT107, the initial dose of CYT107 was increased to 10 μg/kg of CYT107 for all subsequent patients who were randomized to the treatment arm. CYT107 was continued for a total of 3 to 4 weeks or until the patient was discharged from the hospital. Patients randomized to placebo received the same volume and dosing frequency of drug vehicle.

### Quantitation of cytokines.

Cytokine kits for TNF-α, IL-6, and IL-10 were obtained from BioLegend and R&D Systems (Supplemental Methods). Assays were performed as per the manufacturer’s instructions and as previously described (31). Cytokines were analyzed by 3 different laboratories, located in the United Kingdom, France, and the United States.

### Development of secondary hospital-acquired infections.

The development of hospital-acquired secondary infections was evaluated by a 3-member panel of experienced intensive care physicians in the United States and a 3-member panel of experienced intensive care physicians in France using the Center for Disease Control criteria for healthcare-associated infections as previously described (31). The panel members were blinded to patient identity and treatment group.

### Statistics.

All study endpoints were analyzed on the intention to treat (ITT) population (patients randomized to CYT107 or placebo). Safety data analysis included all patients who received at least 1 injection of CYT107. Categorical outcomes were evaluated using Fisher’s exact test. Continuous outcomes and especially ALC were analyzed with linear mixed models for repeated measures incorporating treatment group, measurement day. Last ALC values were analyzed by 2-tailed *t* test for unequal variances. Survival, ICU, and Hospital length of stay were analyzed by Kaplan-Meier curve and a log-rank (Mantel-Cox) test to compare the curves. Note that patients who died during their ICU or hospital stay were censored at day 45 even if they died prior to that day. The ratio of the death rates with associated *P* value is based on Poisson distribution. Statistical analysis was performed in the MedCalc software suite (MedCalc Software Ltd.); however, Log-Rand (Mantel-Cox) was performed in GraphPad Prism 10.3.1.

### Study approval.

The study was conducted at major academic sites in the United Kingdom, France, Brazil, and the United States; trial registrations NCT04379076, NCT04426201, NCT04442178, NCT04407689; and NCT04927169 (https://clinicaltrials.gov). The regulatory agencies at each country (FDA/United States, MHRA/United Kingdom, ANSM/France, CONEP/Brazil) as well as centralized IRBs/Ethical Committees or local committees when applicable approved the study. United Kingdom regulatory approval nos. include Eudract, 2020-001786-36; ISRCTN, ISRCTN15913068. French regulatory agency (ANSM) approval no. is 2020-001573-78. French Ethical Committee approval no. is 20-36/SI 20.04.14.45420. Brazil CONEP approval no. is 37281020.91001.5505. In the United States, approval nos. are MOBAP1138 (Missouri Baptist Hospital, St. Louis, Missouri, USA), IRB2020000924 (University of Florida, Gainesville, Florida, USA), and IRB 3 20-938 (Cleveland Clinic, Cleveland, Ohio, USA).

All patients or their legal representatives gave written informed consent prior to study inclusion.

### Data availability.

Data used for all analyses in the present study are included in the Supporting Data Values file. Patient information is deidentified.

## Author contributions

MSH, BF, KER, CG, SP, TD, RS, SB, LLM, CY, VV, MM, MVDB, and RH conceived and designed the study. MM, AHW, J Blood, FB, RJ, and RH analyzed data and maintained databases. MSH, BF, KER, CG, SP, TD, RS, GA, DS, RSM, NJA, J Blood, J Bosanquet, TB, SB, VV, and FDP provided clinical management and site coordination. All authors reviewed the manuscript and discussed results.

## Supplementary Material

Supplemental data

ICMJE disclosure forms

Supporting data values

## Figures and Tables

**Table 1:**
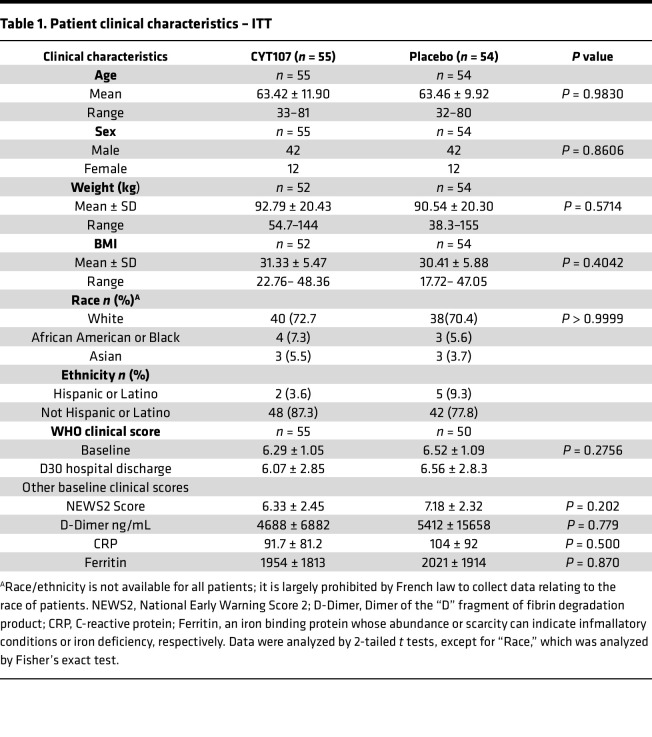
Patient clinical characteristics – ITT

**Table 2:**
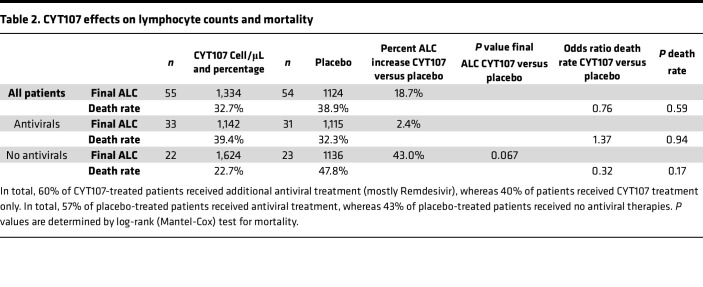
CYT107 effects on lymphocyte counts and mortality

**Table 3:**
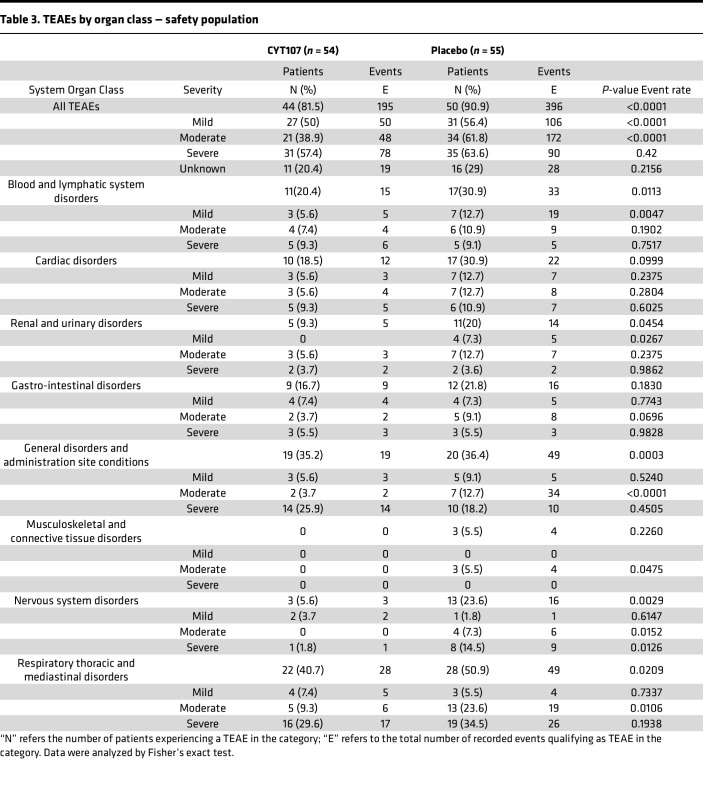
TEAEs by organ class — safety population

**Table 4:**
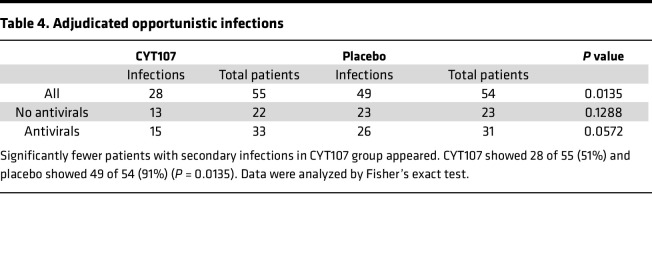
Adjudicated opportunistic infections

**Table 5:**
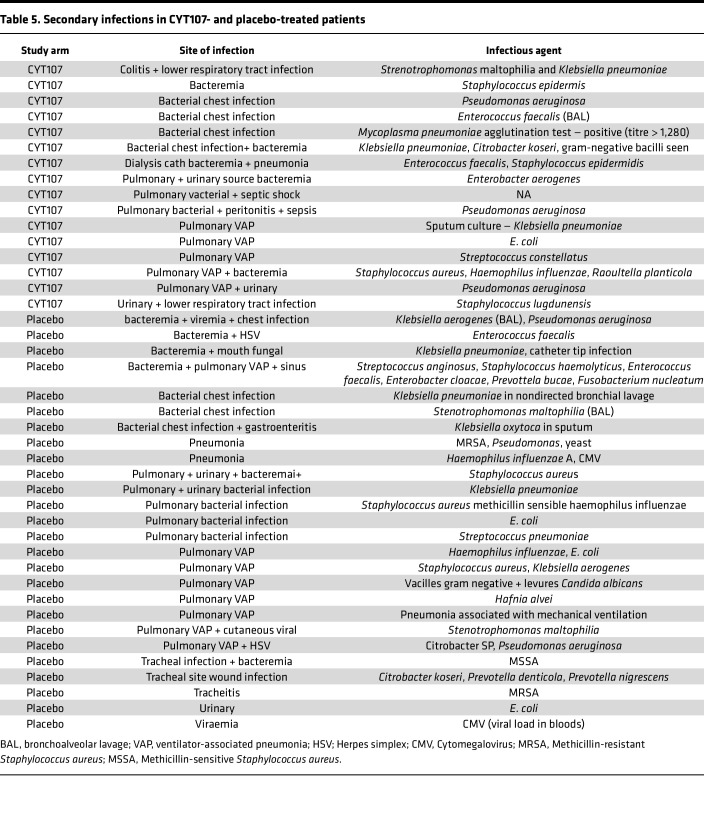
Secondary infections in CYT107- and placebo-treated patients

## Version 1 02/04/2025

In-Press Preview

## Version 2 03/24/2025

Electronic publication
