# Supplementary material for: A randomized, double-blind, placebo-controlled trial of IL-7 in critically ill patients with COVID-19
Source: JCI Insight. 2025 Feb 4;10(6):e189150. doi: 10.1172/jci.insight.189150 (PMC11949036; doi:10.1172/jci.insight.189150)
Supplement: Supplemental data [file jciinsight-10-189150-s179.pdf]

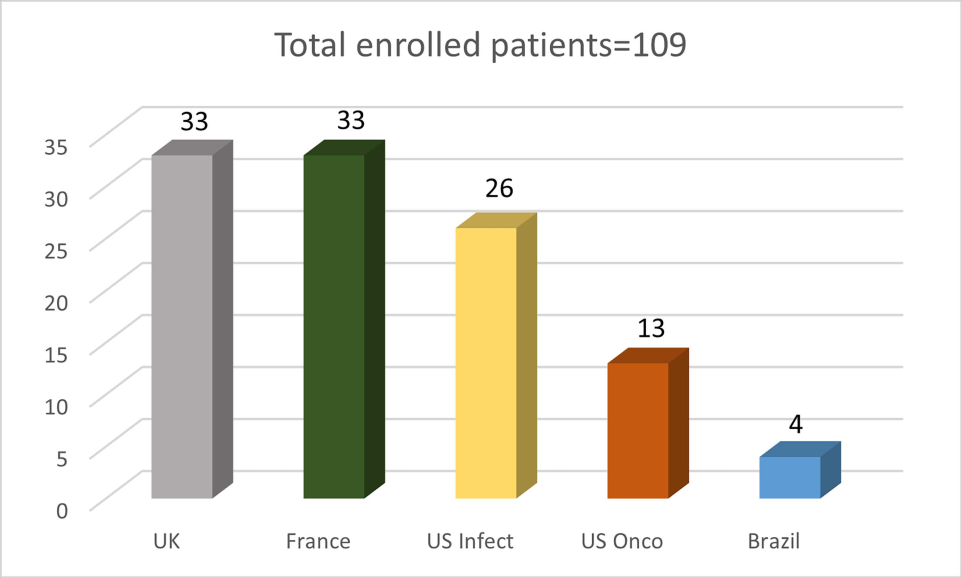
Total enrolled patients=109
35
30
25
20
15
10
5
0
33
33
26
13
4
UK
France
US Infect
US Onco
Brazil

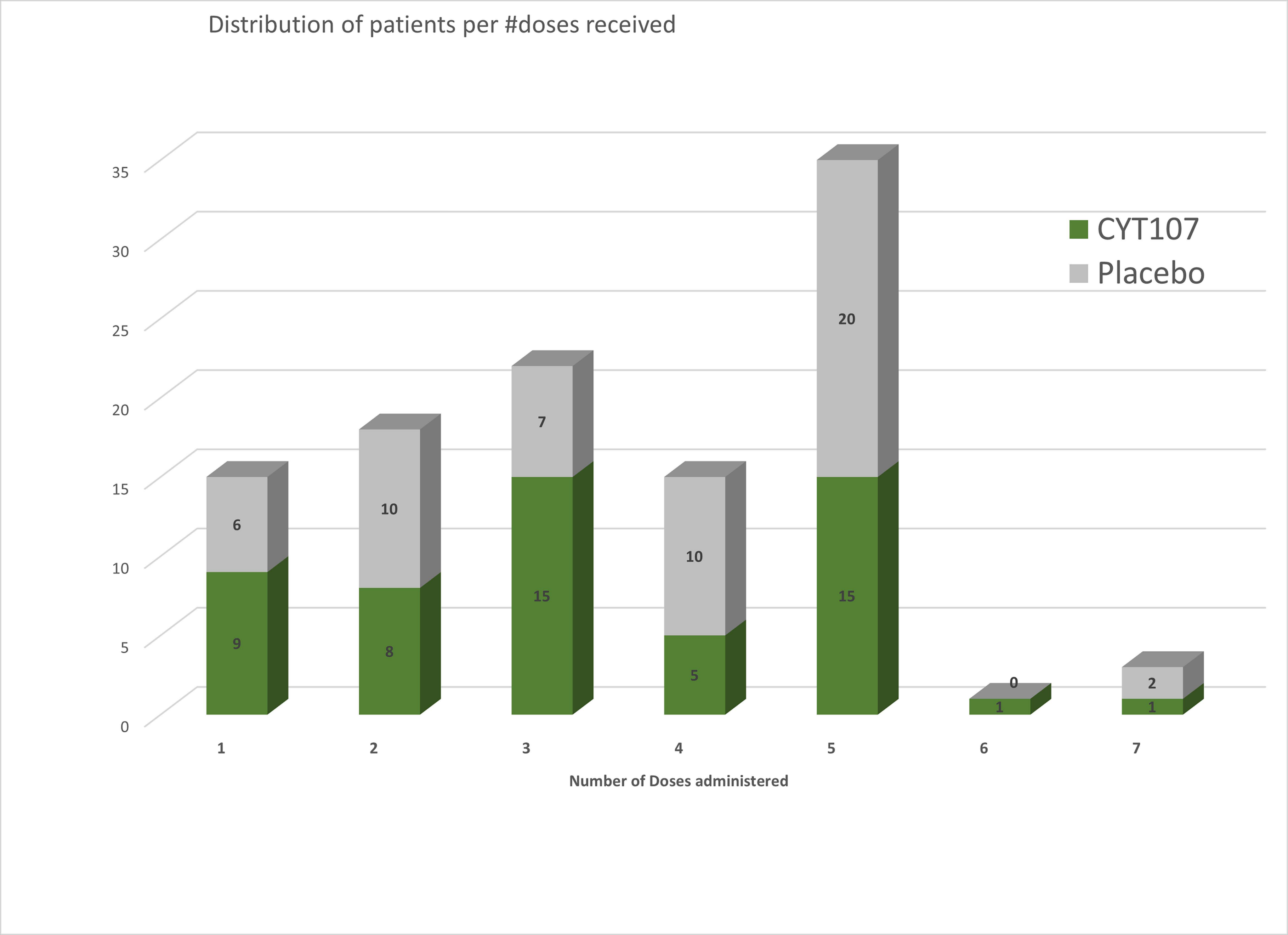
Distribution of patients per #doses received
35
30
25
20
15
10
5
0
CYT107
Placebo
6
9
10
8
7
15
10
5
20
15
0
1
2
1
1
2
3
4
5
6
7
Number of Doses administered

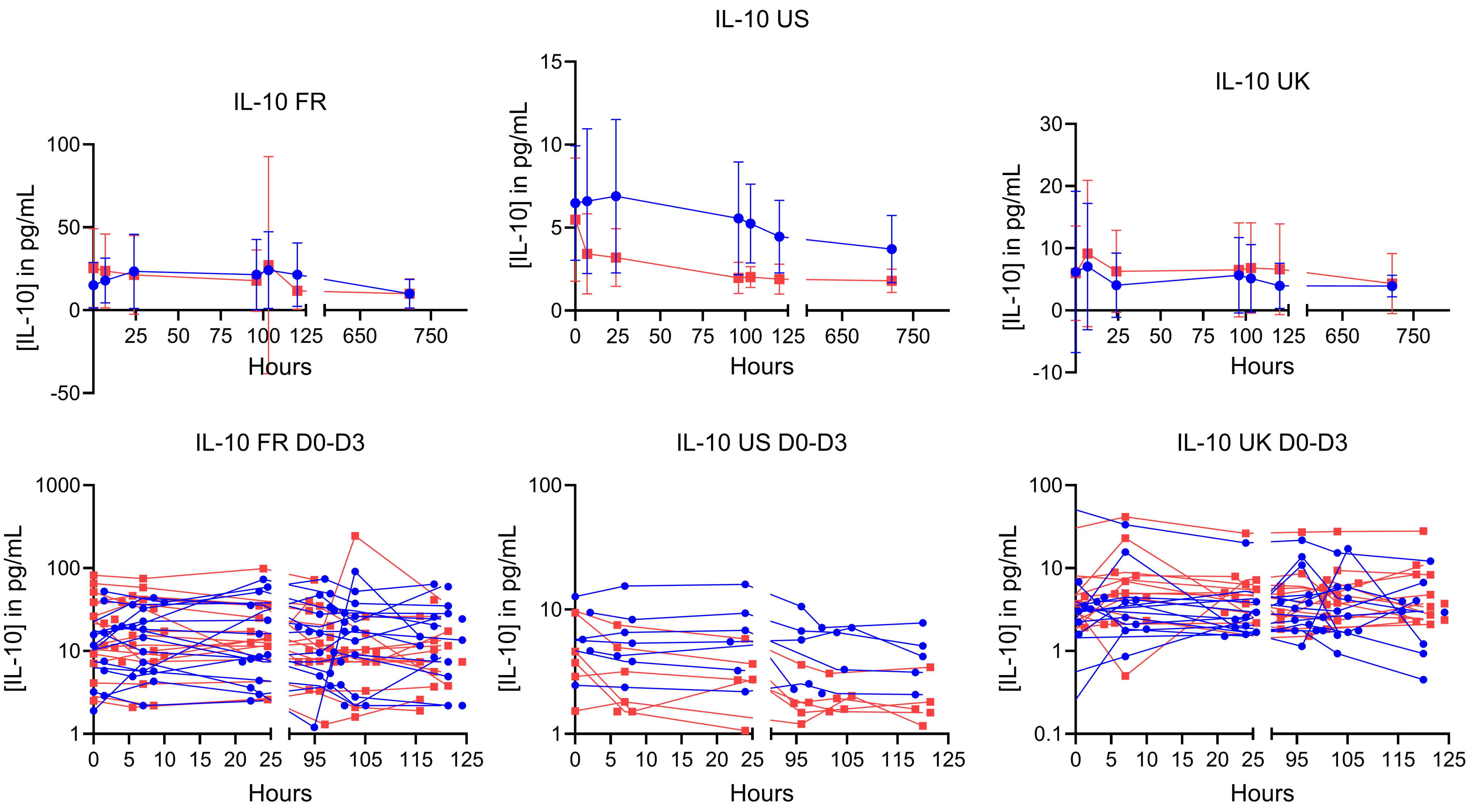

Blue=CYT107
Red=Placebo

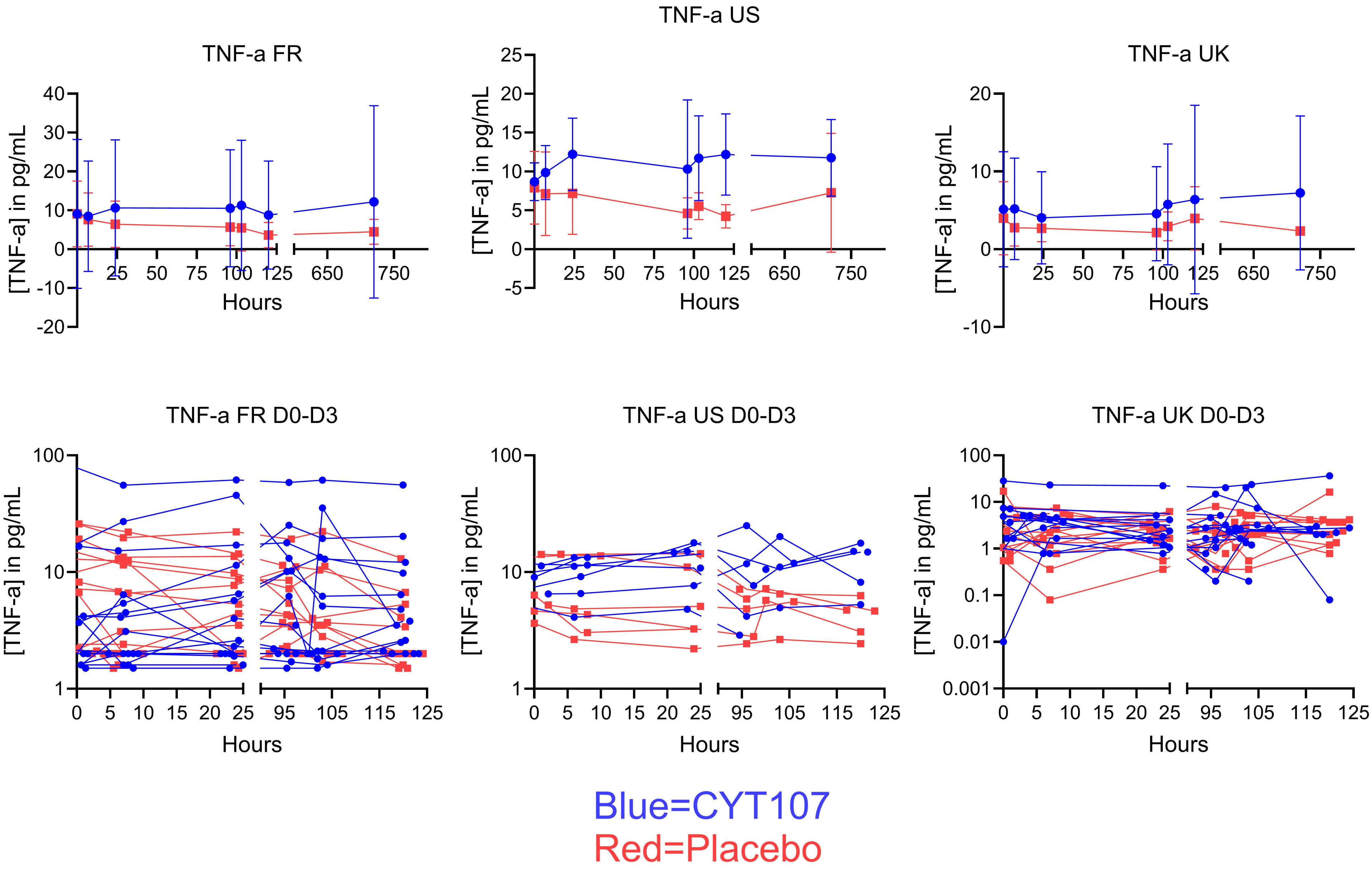
TNF-a US
TNF-a FR
TNF-a UK
[TNF-a] in pg/mL
Hours
TNF-a FR D0-D3
TNF-a US D0-D3
TNF-a UK D0-D3
Blue=CYT107
Red=Placebo

Supplemental Table 1:

	All Patients			Anti-Virals			No Anti-Virals		
	CYT107	Placebo	p-value	CYT107	Placebo	p-value	CYT107	Placebo	p-value
Baseline WBC	9.39 (0.56)	9.98 (0.75)	0.9719	10.10 (0.82)	9.50 (1.09)	0.2892	8.29 (0.60)	10.61 (0.97)	0.0955
Baseline Neutrophils	8.42 (0.56)	8.65 (0.72)	0.8364	8.87 (0.78)	8.30 (0.99)	0.2892	7.71 (0.76)	9.13 (1.05)	0.2598
Baseline Monocytes	0.45 (0.08)	0.44 (0.05)	0.6600	0.48 (0.06)	0.49 (0.07)	0.7556	0.40 (0.06)	0.37 (0.04)	0.8660
Baseline Lymphocytes	0.61 (0.03)	0.51 (0.03)	0.0160	0.61 (0.03)	0.53 (0.3)	0.1105	0.610 (0.05)	0.49 (0.04)	0.0663

Reported values are k/cumm^3 and represent the mean (Standard Error of the Mean) of the relevant study populations. P-values are derived from the Mann-Whitney test. Abbreviations: WBC-White Blood Cells

Supplemental Table 2:

Pts number	Average Duration of antiviral Treatment(days)	SD	Confid Interval ±	MEDIAN	p-value
REMDESIVIR + CYT107 = 31	5,32	3,44	± 6,88	5	
REMDESIVIR + PBO = 27	9,37	12,76	± 25,52	5	P=0,095
ACICLOVIR analogs + CYT107 = 6	31,3	21,2	± 42,4	45	
ACICLOVIR analogs + PBO = 11	23,64	18,2	± 36,4	26	P=0,44

Supplemental Table 3:

Treatment<br>Subject Number	Primary Cause Of Death	Related<br>PI assessment	Related<br>PhV final assessment
CYT107	Pulmonary fibrosis	Not related	No reasonable possibility
CYT107	Pulmonary air leakage	Unlikely to be related	No reasonable possibility
CYT107	Coronavirus pneumonia	Unlikely to be related	No reasonable possibility
CYT107	Multiple organ dysfunction syndrome	Not related	No reasonable possibility
CYT107	COVID-19 pneumonia	Unlikely to be related	No reasonable possibility
CYT107	Acute respiratory failure	Not related	No reasonable possibility
CYT107	Septic shock	Not related	No reasonable possibility
CYT107	Multiple organ dysfunction syndrome	Not related	No reasonable possibility
CYT107	Shock	Not related	No reasonable possibility
CYT107	Respiratory failure	Not related	No reasonable possibility
CYT107	Multiple organ dysfunction syndrome	Not related	No reasonable possibility
CYT107	Multiple organ dysfunction syndrome	Unlikely to be related	No reasonable possibility
CYT107	Multiple organ dysfunction syndrome	Not related	No reasonable possibility
CYT107	Multiple organ dysfunction syndrome	Not related	No reasonable possibility

Treatment<br><br>Subject Number	Primary Cause Of Death	Related<br><br>PI assessment	Related<br><br>PhV final assessment
CYT107	Acute Respiratory failure<br><br>Pneumonia	Unlikely to be related<br><br>Unlikely to be related	No reasonable possibility<br><br>No reasonable possibility
CYT107	Cardiac arrest	Not related	No reasonable possibility
CYT107	Multiple organ dysfunction syndrome	Not related	No reasonable possibility
CYT107	Acute Respiratory failure	Not related	No reasonable possibility
Placebo	Pulmonary fibrosis	Not related	No reasonable possibility
Placebo	Respiratory arrest	Not related	No reasonable possibility
Placebo	Hemorrhagic stroke	Not related	No reasonable possibility
Placebo	Acute respiratory distress syndrome<br><br>Pulmonary fibrosis	Not related<br><br><br><br>Unlikely to be related	No reasonable possibility<br><br>No reasonable possibility
Placebo	Respiratory Failure<br><br><br><br>Cardiac arrest	Unlikely to be related<br><br><br><br>Potentially-Possibly related	Not related<br><br><br><br>No reasonable possibility
Placebo	Multiple organ dysfunction syndrome	Unlikely to be related	No reasonable possibility

Treatment<br><br>Subject Number	Primary Cause Of Death	Related<br><br>PI assessment	Related<br><br>PhV final assessment
Placebo	Shock hemorrhagic	Not related	No reasonable possibility
Placebo	Hypoxia	Not related	No reasonable possibility
Placebo	Pneumonia	Not related	No reasonable possibility
Placebo	COVID-19 pneumonia	Not related	No reasonable possibility
Placebo	Multiple organ dysfunction syndrome	Not related	No reasonable possibility
Placebo	Multiple organ dysfunction syndrome	Not related	No reasonable possibility
Placebo	Multiple organ dysfunction syndrome	Not related	No reasonable possibility
Placebo	Cardiac arrest	Not related	No reasonable possibility
Placebo	Acute respiratory failure<br><br>Shock<br><br>Acute kidney injury	Unlikely to be related<br><br>Potentially-possibly related<br><br>Potentially-possibly related	Not related<br><br>No reasonable possibility<br><br>Reasonable possibility
Placebo	Respiratory failure	Potentially-possibly related	No reasonable possibility

SUPPLEMENTAL METHODS

INCLUSION CRITERIA

In order to be eligible to participate in this Study, an individual must meet all of the following criteria:

1. A written, signed informed consent, or emergency oral consent, by the patient or the patient's legally authorized representative, and the anticipated ability for participant to be re-consented in the future for ongoing Study participation
2. Men and women aged ≥ 25 – 80 (included) years of age
3. Hospitalized patients with one absolute lymphocyte count (ALC) ≤ 1000 cells/mm^3 collected at baseline or no more than 72 hours before baseline
4. Hospitalized patients with hypoxemia requiring oxygen therapy at ≥2L per minute nasal cannula or greater to keep saturations >90%, non-invasive positive pressure ventilation (e.g., BIPAP) for respiratory failure
5. Confirmed infection with COVID-19 by any acceptable test available/utilized at each site
6. Willingness and ability to practice contraception regardless of the gender of the patient during 5 month after last drug exposure

The **co-morbidities** affecting a COVID-19 patient should be recorded (Obesity, diabetes, hypertension, and chemotherapies or immunotherapies for cancer which were completed more than 3 months ago. Patients with these co-morbidities are eligible for the Study and are **included** (except for BMI>40).

As we anticipate more frequent use of new anti-viral drugs in the coming weeks the administration of such drugs should be recorded. Patients who have received or are receiving such drugs for COVID-19 are eligible for the Study and these patients are **included.** As we anticipate ongoing use and testing of **anti-inflammatory drugs** in the coming weeks, the administration of such drugs should also be recorded. Patients who have received or are receiving such drugs for COVID-19 are eligible for the Study and are **included**, although treatment with such drugs should preferably be minimized.
For Patients receiving any agent with immune suppressive effects, **such as** steroids and/or anti-IL6R treatments like Tocilizumab or Sarilumab or anti-IL-1 treatment like Anakinra, these agents should preferably be minimized, **but do not prevent from enrollment**. Recent data from the RECOVERY study showing a benefit in patients with a CRP (C-reactive protein) ≥75 mg/L.

EXCLUSION CRITERIA

An individual who meets any of the following criteria will be excluded from participation in this Study:

1. Pregnancy or breast feeding
2. ALT and/or AST > 5 x ULN
3. Known, active auto-immune disease;
4. Ongoing cancer treatment with chemotherapy / immunotherapy or any cancer therapy within last 3 months and/or ongoing
5. Patients with past history of Solid Organ transplant
6. Active tuberculosis, uncontrolled active HBV or HCV infection, HIV with positive viral load

7. Patients whose respiratory condition is showing significant deterioration as indicated by:
    a. Requirement for a persistent or sustained increase in inspired oxygen concentrations of 20% or more over the past 24 hours to maintain SpO2 at greater than or equal to 88% (this 20 % limit does not apply to O2 delivered by nasal canula)
    b. need for invasive mechanical ventilation
8. Patients with chronic kidney dialysis
9. Patients with a SOFA score ≥ 9 at baseline
10. Patients with a BMI > 40
11. Patients with baseline Rockwood Clinical Frailty Scale ≥ 6 at Hospital admission (*assessed as patient or proxy 4-week recall of chronic health and frailty status prior to COVID infection)*
12. Patients showing a recent increase of the NEWS2 score by more than 6 points during the screening/ baseline period
13. Patients under guardianship

ONCOLOGY COHORT – INCLUSION CRITERIA

In order to be eligible to participate in this Study, an individual must meet all of the following criteria:

1. A written, signed informed consent, or emergency oral consent, by the patient or the patient's legally authorized representative, and the anticipated ability for participant to be re-consented in the future for ongoing Study participation
2. Patients receiving active or recent chemotherapy or immunotherapy (within 6 months) for cancer (and/or)
3. Patients who have received hematopoietic stem cell transplantation (for a diagnosis other than lymphoma) within the past 1 year (and/or)
4. Patients who received CAR-T cell therapy within the past 1 year (but not within last 30 days- see also exclusion criteria number 6 & 7) (and/or)
5. Patients receiving hormonal therapy for cancer (and/or)
6. Patients who have undergone surgery or radiotherapy for cancer within the past 6 months (or)
7. Patients with newly diagnosed (biopsy proven) malignancy who have not yet received cancer treatment but get COVID pneumonia in the interim (Incl. Criteria 11)
8. Men and women aged ≥ 25 – 80 (included) years of age
9. Hospitalized patients with one absolute lymphocyte count (ALC) ≤ 1000 cells/mm^3 collected at baseline or no more than 72h before baseline. (Baseline time is decided by the investigator independently of the initial hospitalization time)From this time point the investigator may choose to further postpone the commencement of IL-7 (CYT107) treatment according to patient's clinical status.
10. Hospitalized patients with hypoxemia requiring oxygen therapy at ≥2L per minute nasal cannula or greater to keep saturations >90%, non-invasive positive pressure ventilation (e.g., BIPAP) for respiratory failure or patients intubated/ventilated for respiratory failure
11. Confirmed infection with COVID-19 by any acceptable test available/utilized at each site
12. Willingness and ability to practice contraception regardless of the gender of the patient during 5 months after last drug exposure

The **co-morbidities** affecting a COVID-19 patient should be recorded (Obesity, diabetes, hypertension, and chemotherapies or immunotherapies for cancer which were completed more than 3 months ago). Patients with these co-morbidities are eligible for the Study and are **included** (except for BMI>40).

As we anticipate more frequent use of new anti-viral drugs in the coming weeks the administration of such drugs should be recorded. Patients who have received or are receiving such drugs for COVID-19 are eligible for the Study and these patients are **included.**

As we anticipate ongoing use and testing of **anti-inflammatory drugs** in the coming weeks, the administration of such drugs should also be recorded. Patients who have received or are receiving such drugs for COVID-19 are eligible for the Study and are **included**, although treatment with such drugs should preferably be minimized.
For Patients receiving any agent with immune suppressive effects, **such as** steroids and/or anti-IL6R treatments like Tocilizumab or Sarilumab or anti-IL-1 treatment like Anakinra, these agents **do not prevent from enrollment**. Recent data from the RECOVERY study showing a benefit in patients with a CRP (C-reactive protein) ≥75 mg/L.

Oncology Cohort – Exclusion Criteria
An individual who meets any of the following criteria will be excluded from participation in this Study:

1. Pregnancy or breast feeding;
2. ALT and/or AST > 5 x ULN
3. Known, active auto-immune disease;
4. Patients with a history of lymphoid malignancy, with the exception of patients with B-cell lymphomas
5. Patients with any malignancy that is present at time of enrollment where treating physician expects life expectancy due to the underlying malignancy to be less than 6 months
6. Patients who received CAR-T cell therapy within the past 30 days or with unresolved cytokine release syndrome (CRS) or immune effector cell-associated neurotoxicity syndrome (ICANS)
7. Patients with unresolved grade ≥ 2 toxicities from prior chemotherapy, immunotherapy, or CAR-T cell therapy
8. Patients with past history of Solid Organ transplant.
9. Active tuberculosis, uncontrolled active HBV or HCV infection, HIV with positive viral load.
10. Hospitalized Patients whose respiratory condition is showing significant deterioration as indicated by:
requirement for a persistent or sustained increase in inspired oxygen concentrations of 20% or more over the past 24 hours to maintain SpO2 at greater than or equal to 88% (this 20 % limit does not apply to O2 delivered by nasal canula)
11. Patients with chronic kidney dialysis
12. Patients with a SOFA score ≥ 9 at baseline
13. Patients with a BMI > 40
14. Patients showing a recent increase of the NEWS2 score by more than 6 points during the screening/ baseline period
15. Patients with hospital admission Rockwood Clinical Frailty Scale ≥ 6 *(assessed as patient or proxy 4-week recall of chronic health and frailty status prior to COVID infection)*
16. Patients under guardianship

Members of the Data Safety Monitoring Board: Dr. Steve Opal, Dr. Elliott Crouser, Dr. Philippe Eggimann, Dr. Peter Pickkers.

Members of the Secondary Infection Adjudication Committee –

-United States and Brazil: Dr. John Mazuski, Dr. Brian Fuller, Dr. Isaiah Turnbull

-France and United Kingdom: Dr. Bruno Abraham, Dr. Marc Clavel, Dr. Valerie Gissot.

Name of Site or Practice Participating in ILIAD 7 Trial of CYT107 in COVID-19

United Kingdom: Manu SHANKAR-HARI, GUY's and ST THOMAS HOSPITAL; Tim FELTON, WYTHENSHAWE HOSPITAL/ MANCHESTER ROYAL INFIRMIRY; Georg AUZINGER, KING'S COLLEGE HOSPITAL; Valerie PAGE, WATFORD GENERAL HOSPITAL; Jane MINTON, ST. JAMES'S UNIVERSITY HOSPITAL; Stephen WRIGHT, ROYAL VICTORIA INFIRMARY AND FREEMAN HOSPITAL; Johnatan HULME, SANDWELL BIRMINGHAM HOSPITAL; Tony WHITEHOUSE UNIVERSITY HOSPITAL BIRMINGHAM; Lisa VINCENT-SMITH, MEDWAY MARITIME HOSPITAL

France: Bruno FRANCOIS & Thomas DAIX, CENTRE HOSPITALIER UNIVRSITAIRE DE LIMOGES; Pierre-Francois DEQUIN, HOPITAL BRETONNEAU; Jean-Paul MIRA, HOPITAL COCHIN-APHP; Laurent ARGAUD & Thomas RIMMELE, HOPITAL EDOUARD HERRIOT; Gregoire MULLER, CENTRE HOSPITALIER REGIONAL D'ORLEANS; Christophe GUITTON, CENTRE HOSPITALIER LE MANS; Jerome ROUSTAN, CENTRE HOSPITALIER MONTAUBAN

United States: Richard HOTCHKISS, WASHINGTON UNIVERSITY SCHOOL OF MEDICINE; Scott BRAKENRIDGE & Phil EFRON, UNIVERISTY OF FLORIDA – GAINESVILLE; Vidula VACHHARAJANI, CLEVELAND CLINIC; Stephen KUPERBERG & Sadia ABBASI, RENAISSANCE SCHOOL OF MEDICINE AT STONY BROOK; Jag SUNDERRAM, RUTGERS ROBERT WOOD JOHNSON MEDICAL SCHOOL; David SRIKER, MISSOURI BAPTIST MEDICAL CENTER ST LOUIS; Ken REMY, UH CLEVELAND MEDICAL CENTER; Robert J. MARTIN & James BOSANQUET & Nitin ANAND, MISSOURI BAPTIST MEDICAL CENTER ST LOUIS; Marcel VAN DEN BRINK, MEMORIAL SLOAN KETTERING CENTER/ CITY OF HOPE; Stephen M. PASTORES, MEMORIAL SLOAN KETTERING CANCER CENTER; Christina GUTIERREZ & Yee CASSIAN, MD ANDERSON

Brazil: Reinaldo SALOMAO, ESCOLA PAULISTA DE MEDICINA – UNIVERSIDADE FEDERAL DE SAO PAULO – UNIFESP; Felipe DAL PIZZOL, HOSPITAL SAO JOSE; Eduardo SPRINZ, HOSPITAL DE CLINICAS DE PORTO ALEGRE DA UNIVERSIDADE FEDERAL DO RIO GRANDE DO SUL – HCPA/UFRGS; Benedito FONSECA, HOSPITAL DAS CLINICAS DA FACULDADE DE MEDICINA DE RIBEIRAO PRETO – USP; Rafael GAIOLLA, UPECLIN – UNESP; Luis CARLOS LOSSO, FINADEN – HOSPITAL EDMUNDO VASCONCELOS – CENTRO DE PESQUISA CLINICA
